# Age-related reversal of spinal excitability during anticipatory postural control

**DOI:** 10.1007/s00421-018-3982-0

**Published:** 2018-09-04

**Authors:** Tibor Hortobágyi, Lajenda E. van de Waardt, Craig D. Tokuno, Wolfgang Taube, Selma Papegaaij

**Affiliations:** 1 Center for Human Movement Sciences, University Medical Center Groningen, University of Groningen, Groningen, The Netherlands; 2 Department of Kinesiology, Brock University, St. Catharines, Canada; 3 Sport and Movement Sciences, Department of Medicine, University of Fribourg, Fribourg, Switzerland

**Keywords:** Aging, H reflex, Posture, Perturbation

## Abstract

**Introduction:**

An internal perturbation of standing balance activates muscles critical for maintaining balance and is preceded by anticipatory postural adjustments (APAs). In healthy younger adults, a measure of spinal excitability in the form of the Hoffmann (H) reflex becomes depressed during APAs but how aging affects the reflex control of APAs is unknown.

**Methods:**

We compared H reflex excitability profiles in the right soleus muscle, indirectly indicating APA, between younger (*n* = 11, age 19–24 years), middle-aged (*n* = 10, age 37–56 years), and older healthy adults (*n* = 11, age 63–78 years). Subjects rapidly raised the right-dominant arm in response to an auditory cue. The H reflex was evoked 120 ms, 100 ms, 80 ms, 60 ms, 40 ms, 20 ms, and 0 ms before as well as 20 ms after the onset of the right anterior deltoid muscle activation. For data processing, each trial was controlled for the corresponding background EMG activity before normalizing the standing data to the data in sitting in the 8 time bins.

**Results:**

All subjects showed a silent period in the soleus background electromyographic activity, suggesting the presence of APA. We found that the stereotypical H reflex depression associated with APAs in younger adults was reduced in middle-aged adults and reversed to facilitation in older adults. The depression occurred in 10 out of 11 younger adults, whereas all 11 older adults exhibited facilitation.

**Conclusion:**

Because APAs are organized at the supraspinal level, we speculate a supraspinal origin of the age-related reflex facilitation during APAs.

## Introduction

A physiological preparatory state precedes voluntary movements. Excitability of cells in the primate primary motor cortex and the supplementary motor area increases 100–400 ms before the start of a voluntary movement (Cheney and Fetz 1980; Evarts 1966). Electroencephalographic (Kornhuber and Deecke 1965) and magnetic brain stimulation studies (Chen et al. 1998; Davey et al. 1998; MacKinnon and Rothwell 2000; Pascual-Leone et al. 1992; Rossini et al. 1988; Starr et al. 1988; Walchli et al. 2017) in humans also revealed increase in the readiness and motor-evoked potentials up to 100 ms before the generation of agonist electromyographic (EMG) activity during self-initiated movements, in reaction time tasks, or platform perturbations. The rise in excitability presumably increases the readiness state of cells involved in the planning and execution of voluntary movements.

An anticipatory postural adjustment (APA) is one strategy used by the central nervous system to prepare for an oncoming postural perturbation. When healthy humans raise one arm while standing, an action often done in daily life, the self-perturbing shoulder flexion torque causes anticipatory activation of rectus abdominis, rectus femoris, and tibialis anterior (Aruin and Latash 1995; Kasai and Kawai 1994; Kawanishi et al. 1999; Belinkii et al. 1967). The muscle activity acts to accelerate the center of mass forwards in anticipation of the posterior destabilizing torque generated at the shoulders. There is also an anticipatory inhibition of erector spinae, biceps femoris, and soleus (Aruin and Latash 1995; Kasai and Kawai 1994; Kawanishi et al. 1999; Belinkii et al. 1967). Specifically, the soleus muscle activity decreases and a ~ 100-ms-long silent period starts ~ 20 ms before and overlaps the anterior deltoid activation. Spinal excitability measured by the Hoffmann (H) reflex during this silent period declines sharply with the reflex depression peaking around the onset of EMG activity in the anterior deltoid (Kasai and Kawai 1994; Kawanishi et al. 1999). Because the reflex depression starts before the onset of anterior deltoid EMG activity, this anticipatory depression is likely to be the result of a preprogrammed descending motor command acting on the motoneuron directly or via Ia inhibitory interneurons, giving rise to a feedforward control of APAs in standing and also during walking (Capaday and Stein 1986; Simonsen et al. 1995).

Healthy aging affects APAs produced by external and internal perturbations. While the typical APA-associated muscle activity occurs in healthy older adults, it can be delayed (Rogers et al. 1992; Woollacott and Manchester 1993; Kanekar and Aruin 2014). For example, when healthy participants were instructed to stop the swing of a pendulum carrying a weight of 5% of body mass with their extended arms, the onset latency of six muscles was 126 ms in younger and 60 ms in older adults before the contact with the pendulum (Kanekar and Aruin 2014). Thus, the APA occurred much closer to the time of impact in older compared with younger subjects, causing a delay in the subsequent compensatory behavioral adjustments. Little is known about the underlying neural mechanisms. For example, it is unclear if older age affects reflex excitability present during a self-perturbation-induced APA. As aging decreases cortical inhibitory processes in general (Papegaaij et al. 2014) and because the age-related reduction in the excitability of inhibitory circuits in the primary motor cortex is accompanied by a decrease in the contribution of Ia afferent input to the activity of muscles controlling posture (Baudry et al. 2015; Papegaaij and Hortobágyi 2017), we hypothesized that age affects reflex excitability associated with APA by reducing the magnitude of depression. In other words, we expected an age-related disinhibition of H reflexes during APAs. For this purpose, we compared the H reflex excitability profile associated with an APA between younger, middle-aged, and older healthy adults.

## Methods

### Participants

Healthy, right-handed (Oldfield 1971) adult volunteers participated in the study and were stratified based on age as younger (19–24 years, *n* = 11), middle-aged (37–56 years, *n* = 10), or older adults (63–78 years, *n* = 11). After a personal or telephone interview, subject candidates filled in a health questionnaire. Participants were excluded if they reported current or past Parkinson’s disease, multiple sclerosis, diabetes, stroke, cardiac dysfunctions, orthopedic and cognitive problems, were institutionalized, or were taking sedatives, hypnotics, antidepressants, and benzodiazepines, could not stand without rest or assistance for 10 min, and had uncorrected vision. Participants completed the Edinburgh Handedness Inventory (Oldfield 1971), Mini Mental State Examination (MMSE) (Folstein et al. 1975), short physical performance battery (SPPB) (Guralnik et al. 1994), and the Short Questionnaire to Assess Health enhancing Physical Activity (SQUASH) (Wendel-Vos et al. 2003). Participants visited the laboratory one time for a 2-h-long session. Each participant signed an informed consent document approved by the University Medical Ethical Committee and the protocol adhered to the principles of the 2013 Declaration of Helsinki.

### Electromyography (EMG)

After shaving and cleaning the appropriate areas of the skin with alcohol, surface EMG electrodes, equipped with 3D accelerometers, were placed on the right anterior deltoid muscle (dominant arm for all subjects) and the right soleus, whence we recorded the background EMG activity (bEMG) and the reflex responses to peripheral nerve stimulation. An electrode was also placed on the right wrist and on the lateral aspect of the right knee to measure 1D linear acceleration in the anterior–posterior direction. The electrodes were 37 mm × 27 mm × 15 mm, 15 g, wireless, pre-amplified (909×) parallel-bar sensors, affixed to the skin with a four-slot adhesive skin interface (Trigno, Delsys Inc., Natick, MA, USA). The electrodes had a bandwidth of 20–450 Hz, channel noise < 0.75 µV, common mode rejection ratio > 80dB. Signals were sampled at 4 kHz by a 16-bit A/D board (Power 1401, CED, Cambridge, UK) and acquired online, displayed, and stored by software installed on a personal computer for offline analysis (Signal v. 5.0, Spike2 v. 7.12, CED, Cambridge, UK).

### Peripheral nerve stimulation

While participants stood with their feet shoulder-width apart, electrical stimuli (1-ms duration) were first delivered by a constant-current stimulator (DS7A, Digitimer, Hertfordshire, UK) to the right posterior tibial nerve in the popliteal fossa with a hand-held unipolar probing pen electrode to find the optimal location to evoke a muscle response (M wave) and the H reflex. After the responses to the stimuli were identified based on consistency of latency and waveform, two pre-gelled unipolar, 8-mm diameter, Ag–AgCl surface electrodes, cathode in the popliteal fossa and the anode above the patella were taped to the skin. An input–output curve for the H reflex and M wave was created by progressively increasing the stimulation intensity in steps of 0.5 mA (3 stimuli per step) until the M wave amplitude reached its maximal value (Mmax) followed by supramaximal stimuli to ensure that the responses had reached a plateau.

### Experimental protocol

**Fig. 1:**
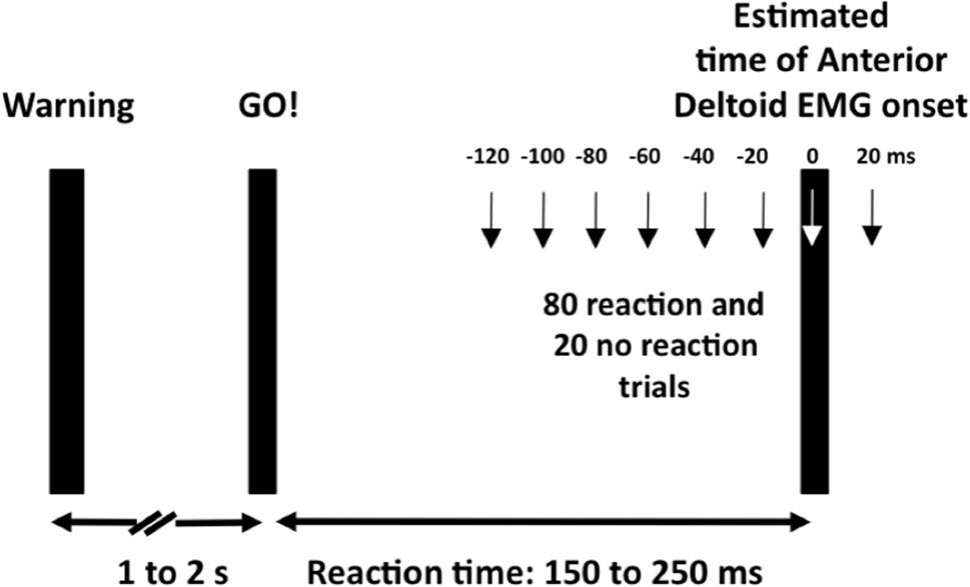
Experimental protocol to determine the effects of age (young, middle-aged, old) and posture (sitting, standing) on a spinal element of the anticipatory posture adjustment, APA. In 20 preliminary trials, we measured subjects’ reaction time in response to an auditory cue after a visual warning signal and also the onset of EMG activity of the anterior deltoid. Based on this estimated interval, we stimulated the posterior tibial nerve to evoke an H reflex in 80 reaction time trials (arm swing) and 40 dummy trials (no arm swing) in 20 ms intervals relative to the estimated anterior deltoid onset in sitting and also in standing. In response to the auditory GO! signal, subjects rapidly swung their right arm from the side of the body to a horizontal position, causing a self-perturbation. The warning signal was a light

Figure 1 shows the experimental arrangement to determine the effects of age (young, middle-aged, old) and posture (sitting, standing) on the spinal element of APA induced by a reactive, rapid arm swing self-perturbation. For each trial, there was a warning light followed 1–2 s later by an imperative GO! auditory cue that prompted subjects to rapidly flex their shoulder and raise the right-dominant arm to the horizontal. In standing, we evoked the H reflex in the right soleus at an intensity corresponding to 20% of Mmax, resulting in an H reflex on the ascending portion of its recruitment curve and—in most participants—a small M wave. In the sitting condition, subjects sat on a chair with their right knee flexed at a position between 100 and 170° while pressing gently with their right foot against a 20 cm tall vertical metal bar fixed in the floor to match the soleus bEMG measured during standing. Subjects viewed a large wall-projection screen, showing the target bEMG activity for the right soleus. We adjusted the stimulation parameters in sitting so that the size of the control H reflex (i.e., no arm swing) was similar to the H reflex measured during standing. We compared the standing data to sitting because unlike in standing, arm swing in sitting does not require an APA in the soleus. We matched the size of the H reflex because a conditioning effect on the H reflex depends on the size of the H reflex relative to Mmax (Crone et al. 1990).

Table 1 summarizes the experimental conditions. Subjects started the experiment by standing quietly for 30 s while we recorded soleus bEMG activity. Subjects then practiced the arm movement in standing without nerve stimulation and practiced, in sitting, to generate the same level of soleus bEMG activity measured previously in standing. From these preliminary trials, we computed reaction time, i.e., the time interval between the GO! signal and the start of shoulder flexion and then custom-programmed for each subject the time of peripheral nerve stimulation relative to the estimated EMG onset of the anterior deltoid.

**Table 1:** Summary of experimental sequence and conditions

Posture	Purpose	Block	Perturbation	Stimulation	No. of trials	Duration (s)
Standing	Measure bEMG		No	No	1	30
Standing	Familiarization		Yes	No	10	80
Standing	Estimate reaction time		Yes	No	20	160
Sitting	Familiarization		Yes	No	10	80
Sitting	Estimate reaction time		Yes	No	20	160
Standing	Recruitment curve		No	Yes	2 per step	300
Standing	Control trials	1a	No	Yes	20	120
Standing	Experimental trials	1b	Yes	Yes	40	320
Any	Rest		No	No	0	120
Standing	Experimental trials	2a	Yes	Yes	40	320
Standing	Control trials	2b	No	Yes	20	160
Any	Rest		No	No	0	120
Sitting	Control trials	3a	No	Yes	20	120
Sitting	Experimental trials	3b	Yes	Yes	40	320
Any	Rest		No	No	0	120
Sitting	Experimental trials	4a	Yes	Yes	40	320
Sitting	Control trials	4b	No	Yes	20	120

‘Experimental trials refer to trials done with a self-perturbing arm swing

‘Control trials refer to trials done without a self-perturbing arm swing

In the main experiment, we evoked 120 H reflexes, each 5–7 s apart, in standing and sitting. Within each postural condition, 40 H reflexes were obtained without arm swing (control) and the remaining 80 were collected at eight different time points − 120 ms, − 100 ms, − 80 ms, − 60 ms, − 40 ms, − 20 ms, 0 ms, and + 20 ms relative to the estimated anterior deltoid EMG onset. Thus, a total of 240 H reflexes were evoked throughout the experiment. H reflexes and M waves were additionally recorded at the start and the end of each posture condition (sitting, standing) to determine changes in the stimulation characteristics as well as other confounding factors such as fatigue or changes in attention throughout the experiment (Table 1, blocks 1a and 2b and blocks 3a and 4b). The main blocks of standing (blocks 1, 2) and sitting (blocks 3,4) were not randomized because older adults, as revealed by pilot expriments, could not have finished the experiment without undue fatigue by standing for the second half of the main experiment.

### Data reduction

Reaction time was defined as the interval between the auditory GO! signal and the onset of arm movement. The peak-to-peak amplitude of Hmax and Mmax were determined from the unrectified EMG signals of the recruitment curves and used to set the stimulation intensity. For the APA trials, we sorted the H reflexes into 20 ms bins relative to the actual anterior deltoid EMG onset and examined the time course of APA from − 120 ms before to 20 ms after deltoid EMG onset. Soleus H reflexes recorded during the APA trials (arm swing) were expressed relative to those recorded during the control trials (no arm swing). This normalization process allowed us to determine whether self-perturbation of posture by a unilateral arm swing modified the size of the H reflex, reflecting facilitatory or inhibitory APAs.

### Statistical analyses

Data are reported as means (± SD). We analyzed the reaction time, wrist acceleration, and knee acceleration data using a group (young, old) and posture (sitting, standing) analysis of variance (ANOVA) with repeated measures on posture. For this analysis we pooled the values across all blocks. In a separate analysis, we checked if the length of the experiment affected reaction time (i.e., fatigue) and used a group (young, old) by time (start, end of experiment) ANOVA with repeated measures on time. For the start of the experiment, the data comprised 20 trials used to estimate reaction time. For the end of the experiment, the data comprised 20 trials of block. We analyzed the APA data by group (young, middle-aged, old), by posture (sitting, standing), by bin (8 bins, − 120 to + 20 ms) ANOVA with repeated measures on posture and bins followed by a Tukey’s post hoc contrast to determine the means that differed at *p* < 0.05. All analyses were done in SPSS version 22 (IBM, Armonk, NY, USA).

## Results

### Behavioral data

Table 2 shows that participants were physically active, highly mobile, and physically and cognitively intact. The group (young, old) and posture (sitting, standing) interactions for reaction time (*p* = 0.394) and wrist (*p* = 0.718) and knee acceleration (*p* = 0.666) (data pooled across all blocks) were not significant. Reaction time in the arm swing task was 13 ms shorter in sitting (166.3 ± 40.67 ms) than standing (179.3 ± 38.33, *p* = 0.043) but not different between age groups (*p* = 0.768, Table 2). Peak linear acceleration measured at the wrist during the self-perturbation arm swing did not differ between sitting (53.6 ± 14.8) and standing (55.3 ± 14.4 m·s^− 2^, *p* = 0.445) and between age groups (*p* = 0.781). The arm swing-induced postural perturbation in terms of peak linear acceleration at the knee joint did not differ between sitting (0.20 ± 0.06 m/s^2^) and standing (0.18 ± 0.08 m/s^2^, *p* = 0.551) and between age groups (*p* = 0.324, Table 2).

**Table 2:** Participant characteristics

	Young	Middle	Old
*n* (M)	11 (5M)	10 (4M)	11 (4M)
Age (year)	21.3 ± 1.6	46.8 ± 5.5	70.4 ± 3.9
Height (m)	1.75 ± 0.07	1.68 ± 0.09	1.72 ± 0.09
Mass (kg)	68.8 ± 12.7	75.7 ± 11.9	70.4 ± 11.4
BMI (kg m^− 2^)	22.3 ± 2.3	26.6 ± 3.0	23.7 ± 2.4
MMSE (/30)	29.8 ± 0.4	29.8 ± 0.6	29.4 ± 1.4
SQUASH	11.7 ± 5.2	10.6 ± 4.3	9.9 ± 3.9
SPPB (/12)	12.0 ± 0.0	11.8 ± 0.6	11.5 ± 0.8
Reaction time (ms)			
Sitting	166.5 34.7	168.6 ± 39.9	168.8 ± 48.9
Standing	169.5 ± 33.5	191.7 ± 39.3	180.0 ± 50.1
Acceleration (m·s^− 2^)			
Wrist			
Sitting	54.0 ± 15.4	55.0 ± 15.2	56.9 ± 12.7
Standing	51.2 ± 17.0	53.4 ± 12.2	56.4 ± 15.2
Knee			
Sitting	0.20 ± 0.11	0.23 ± 0.11	0.13 ± 0.04
Standing	0.21 ± 0.07	0.17 ± 0.05	0.21 ± 0.07

Values are mean ± SD

*BMI* body mass index, *MMSE* mini mental state examination, *SQUASH* Short Questionnaire to Assess Health enhancing Physical Activity in total score of the number of minutes physical activity times the intensity of physical activity, *SPPB* short physical performance battery

In a separate analysis we checked using the reaction time data whether fatigue affected the APA findings. The group (young, old) by time (start, end of experiment) interaction was not significant (*F* = 0.88, *p* = 0.729). The reaction time measured in the block (last 10 of 20 trials) at the start of the experiment (3rd row under the header row in Table 1) was 171.7 ms (± 31.6) and it was 167.5 ms (± 30.2) in block 4a (last 10 of 20 trials) during standing in the young. The corresponding values in the old participants were 169.4 ms (± 49.3) and 166.9 ms (± 47.3, *p* = 0.587).

### Effects of age and posture on Hmax and Mmax

We ascertained the presence of APA by analyzing the soleus bEMG activity in trials with and without the self-perturbing arm swing in the absence of peripheral nerve stimulation. We found a 85% depression (i.e., silent period) in the mean rectified and smoothed EMG activity (*p* = 0.003) in the ~ 80 ms window before arm acceleration onset, confirming the presence of an APA (Kasai and Kawai 1994; Kawanishi et al. 1999). However, the soleus bEMG in sitting with (6.6 ± 2.37 µV) and without arm swing (6.8 ± 2.11) did not differ (*p* = 0.391) and there also was no difference in bEMG between the three age groups (*p* = 0.519).

The group (young, middle, old) by posture (sit, stand) interaction for Hmax extracted from the recruitment curve was not significant (*p* = 0.509). Hmax was smaller in sitting (0.70 ± 0.83 mV) than standing (0.92 ± 0.87 mV, *p* = 0.003). Hmax differed between the three age groups: largest in younger (1.63 ± 0.90 mV), followed by middle-aged (0.57 ± 0.46 mV), and older adults (0.19 ± 0.15 mV, all different, *p* = 0.001). The Mmax was largest in younger (3.1 ± 1.24 mV), followed by middle-aged (1.9 ± 0.66 mV), and older adults (1.0 ± 0.43 mV, *p* = 0.001) but Mmax was similar in sitting and standing (*p* = 0.645). For the Hmax/Mmax ratio there was no group by posture interaction (*p* = 0.248), but the ratio was smaller in sitting than standing (30.9 ± 28.71 vs. 41.2 ± 26.44; *p* = 0.025). The ratio in the younger group (54.2 ± 20.03) was higher than the ratio in the middle-aged (30.9 ± 26.24) and older group (22.7 ± 27.33, age main effect, *p* = 0.004).

### Effects of age and posture on APA

The number of trials from which we determined APA did not differ between the two postures (sitting: 7.3 ± 3.61; standing: 7.2 ± 4.08, *p* = 0.981) and between age groups (young: 7.3 ± 3.8; middle-aged: 7.2 ± 3.93; old: 7.2 ± 3.93, *p* = 0.963) but differed (*p* = 0.001) between the eight time bins. Averaged across the two postures and three age groups, the number of trials ranged from 4.7 (± 3.61) in the − 120 ms bin to 8.7 (± 4.01) in the − 80 ms bin.

**Fig. 2:**
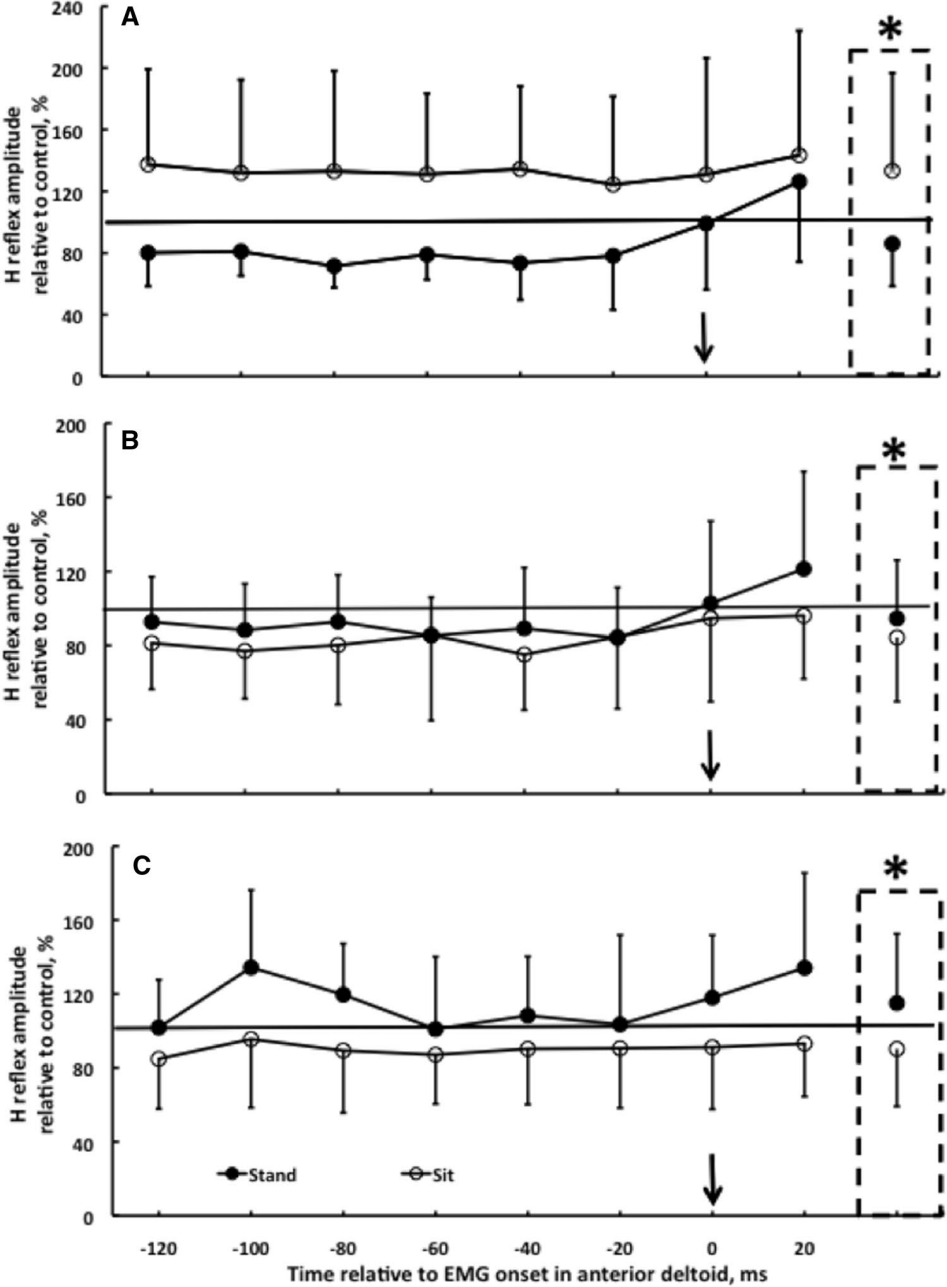
Effects of age and posture on the time course of H reflex in the right soleus muscle in sitting (open symbols) and standing (filled symbols) in younger (**a**, *n* = 11), middle-aged (**b**, *n* = 10), and older adults (**c**, *n* = 11, group by posture by time bin interaction, *p* = 0.818). The H reflex, measured during self-perturbation produced by swinging the right arm from vertical to horizontal, is expressed relative to the size of the control H reflex recorded in trials without a self-perturbation in the two postures. Values above and below the horizontal line, respectively, correspond to a facilitation and inhibition of the H reflex relative to control. The vertical arrow denotes time 0, the onset of the right anterior deltoid EMG activity. The dash-lined boxes denote the group (young, middle-aged, old) by posture (sitting, standing) interaction (*p = 0.002)

Figure 2 shows the H reflex data during APA. Of the group by posture by bin 3-way interaction (*p* = 0.818), the salient finding was the group (young, middle-aged, old) by posture (sitting, standing) interaction (*p* = 0.002). Compared to the control trials without arm swing, younger adults showed an average H reflex depression of 14% across all bins, with a peak depression of 29% in the − 80-ms bin in standing. In contrast, in sitting there was 33% facilitation across all eight bins compared to trials without arm swing (Fig. 2a, *p* < 0.05 standing vs. sitting). This pattern became somewhat reversed in middle-aged subjects, as there was 5% facilitation in standing and a 16% depression in sitting during trials involving APAs (Fig. 2b). The younger pattern became fully reversed in the older group, as, instead of inhibition, there was a 15% facilitation of the soleus H reflex in standing and a 10% inhibition in sitting in trials with arm swing compared to trials without arm swing (Fig. 2c, *p* < 0.05 standing vs. sitting). Post hoc analysis revealed that the depression in standing in young (filled symbol in Fig. 2a) was different from the facilitation (filled symbol in Fig. 2c) observed in standing in old adults (*p* < 0.05).

The reported APA data were acquired so that the bEMG and the H reflex size in sitting matched the values in standing. Thus, the size of the control H reflex (i.e., trials without an arm swing) was 0.25 ± 0.12 mV (*n* = 32) in sitting and 0.24 ± 0.16 mV (*n* = 32) in standing (*p* = 0.478). The group (young, middle-aged, old) by posture (sit, stand) ANOVA revealed that the recording conditions were stable during the course of the experiment because the size of the M waves accompanying the H reflexes in the APA trials were not different between sitting (present in *n* = 26, *p* = 0.662) and standing (present in *n* = 24, posture main effect: *p* = 0.792). Specifically, during the APA trials the size of the M wave averaged across the eight bins was 0.74 mV (± 0.31) in sitting and 0.80 mV (± 0.36) in standing in young subjects. In middle-aged adults, the values were 0.49 mV (± 0.15, sitting) and 0.45 mV (± 0.15, standing). In old adults, the values were 0.37 mV (± 0.15, sitting) and 0.36 mV (± 0.16, standing). There was an age main effect (young: 0.77 ± 0.33 mV; middle-aged: 0.47 ± 0.16 mV; old: 0.37 ± 0.15 mV, *p* = 0.028) so that young adults’ values were higher (*p* < 0.05, Tukey’s post hoc) than the values in the other two age groups. The soleus bEMG measured during 30 s of quiet standing was similar (*p* = 0.519) in the three age groups (young: 7.4 ± 2.80 µV; middle-aged: 6.6 ± 1.9 µV; old: 6.3 ± 2.4 µV) and the values in sitting were numerically nearly identical as a result of matching.

We observed no drift in the H reflexes from start to end of the experiment, as the size of the H reflex in a block of 20 trials at the start and end remained unchanged in sitting (*p* = 0.720, between blocks 3a and 4b) and standing (*p* = 0.299, between blocks 1a and 2b, Table 1). Thus, neither the H reflex data nor the reaction time data (presented in the Behavioral section of the “Results”) suggest evidence for the presence of fatigue.

## Discussion

Under appropriate experimental controls, we found that the stereotypical H reflex depression associated with an APA in younger adults (age ~ 21) was reduced in middle-aged adults (age ~ 47) and reversed to facilitation in healthy older adults (age ~ 70). These data add to the emerging view of age-related impairments in the inhibitory neural control of voluntary movement (Baudry et al. 2015; Levin et al. 2014; Papegaaij and Hortobágyi 2017).

The average (14%) and peak (29%) depression in the H reflex (Fig. 2) during APAs qualitatively agrees with the pattern of depression reported previously (Kasai and Kawai 1994; Kawanishi et al. 1999). However, the depression we observed was about half of what was reported in those previous studies despite using the same arm-lifting task. The reason for this difference in APA magnitude is unclear but may be related to the different ways the reflex amplitude was normalized. In the present study, the reference H reflex was obtained during sitting while in the previous study the comparison H reflexes were elicited while standing with back support (Kawanishi et al. 1999). Using the arm swing model, the H reflex depression occurred in 17 out of 26 or 65% of healthy younger subjects in a previous study (Kawanishi et al. 1999) while we observed depression in 10 out of 11 (i.e., 90%) younger subjects. In contrast and most interestingly, all 11 older adults exhibited reflex facilitation. Independent of age, in the present study all 32 subjects exhibited a depression in the soleus bEMG upon which the H reflex was elicited. Thus, the H reflex facilitation in older and the depression in younger subjects occurred while the soleus bEMG activity revealed a silent period. It is therefore unlikely that the level of bEMG differentially influenced the H reflex in young and old. Consequently, these data rather suggest a clear age-related modification of the Ia afferent transmission during APAs. One likely mechanism that does not affect the bEMG activity would be presynaptic inhibition. This idea would fit with the model presented previously (Kawanishi et al. 1999). This model proposes that an early part of the inhibition associated with APAs emerges from descending pathways onto inhibitory interneurons that directly reduce the excitability of the motoneuron pool. This effect may have led to the reduced bEMG that we observed in all three age groups. Apart from this early component, Kawanishi et al. describe a late inhibitory mechanism that seems to primarily affect Ia presynaptic inhibition. Based on the present data, it might be assumed that aging specifically affects this second component of inhibition. Based on previous data we assume that supraspinal centers adjust the level of presynaptic inhibition during stance (Katz et al. 1988; Nielsen and Petersen 1994). This offers the advantage of selectively blocking the influence of peripheral feedback onto the motoneurons but at the same time maintaining control of the very same motoneurons via descending direct and indirect projections.

The functional relevance of the soleus silent period and the age-related conversion of H reflex depression to an abolishment of the depression at middle age and conversion of the depression to facilitation in older age is unclear. APAs are needed to generate subsequent compensatory movements that correct postural instabilities caused by perturbations (Aruin and Latash 1995; Kanekar and Aruin 2014). Instead of relying on a feedforward mechanism through APAs to correct perturbation-induced movements (Desmurget and Grafton 2000; Fautrelle and Bonnetblanc 2012), older adults tend to use a reactive strategy because the pre-activation of muscles needed to correct the movements is substantially delayed (Aruin and Latash 1995; Kanekar and Aruin 2014; Kubicki et al. 2012a). Our data speculatively imply that the age-related facilitation of the synapse between the Ia afferent and motoneuron contributes to this reactive strategy by stiffening the ankle joint through the coactivation of agonist and antagonist muscles via a reduction of presynaptic inhibition and an increase of descending command to these muscles (Baudry and Duchateau 2012; Hortobágyi and Devita 2006, 2000; Kido et al. 2004). However, since previous studies and the present study did not measure ankle movements in conjunction with H reflex measurements, the functional role of the soleus silent period in APAs remains elusive (Rothwell 1994). While interventions designed to improve older adults’ APAs did favorably modify the activation and latency patterns of putative muscles involved in APAs and the subsequent compensatory behaviors (Kanekar and Aruin 2014; Kubicki et al. 2014, 2012b), future interventions should provide more direct evidence that the silent period of the soleus and the newly observed H reflex facilitation in older adults’ APAs can be favorably modified and that such neural changes underlie the compensatory behavioral changes.

A limitation of the present and past APA H reflex studies is a lack of examination of the association between any behavioral postural adjustment and the modulation of the H reflex and soleus silent period. Such studies are needed because interventions can, remarkably, enhance APAs by making muscles respond sooner to perturbations. However, it is unclear if such earlier activation onsets are related to spinal or cortical excitability. We focused the current experiment on the H reflex as an outcome, which we could record only in one muscle. We thus cannot tell if the conclusions are true for other muscles as well. A limitation in conjunction with the H reflex measurements in the present study is that while the size of the H reflex and the accompanying M wave was each matched between the control condition in sitting and the experimental condition in standing but such matching was not possible between age groups, which could affect the results (Crone et al. 1990). However, this limitation was minimized by the observation that while the H reflex measured in the APA trials was modulated differently between age groups, the accompanying M waves were not. Also, the experiments lack biomechanical measurements other than arm and knee accelerations. We did not specifically check for age-related reductions in visual and auditory acuity, which if present could affect APAs. For ethical reasons it was not possible to have older adults perform more trials, limiting the number of observations in certain time bins.

In conclusion, after controlling for numerous experimental parameters, we found that the stereotypical H reflex depression associated with an APA in younger adults (age 19–24 years) was reduced already in middle-aged adults (age 37–56 years) and reversed to facilitation in healthy older adults (age 63–78 years). We speculate a supraspinal effect contributing to the age-related reflex facilitation during APAs.

## Abbreviations

A/D: Analog to digital conversion
APA: Anticipatory postural adjustments
CED: Cambridge Electronics
bEMG: Background EMG activity
EMG: Electromyography
H reflex: Hoffmann reflex
MMSE: Mini mental state examination
M wave: Muscle compound action potential
SPPB: Short physical performance battery
SQUASH: Short Questionnaire to Assess Health enhancing Physical Activity
